# What Laboratories Can Do to Make Space for People with Disabilities

**DOI:** 10.1021/acscentsci.4c00486

**Published:** 2024-03-29

**Authors:** Krystal Vasquez

“If anyone were to borrow a wheelchair for a day, I think they’d be pretty surprised about how little they could do in actual physical lab space,” says Paul Bracher, a chemistry professor at Saint Louis University.

Bracher, who uses a wheelchair, says workbenches and fume hoods are usually too high off the ground for him to use from a seated position. Many laboratories also have narrow, cluttered walkways that can be impossible for him to navigate.

By now, Bracher has come to expect that most laboratories he visits won’t be accessible to him, but nondisabled scientists are often taken aback. Emrys Travis, a disability and accessibility specialist at the UK’s Royal Society of Chemistry, has heard people say, “Surely it’s illegal to not have wheelchair access.”

**Figure:**
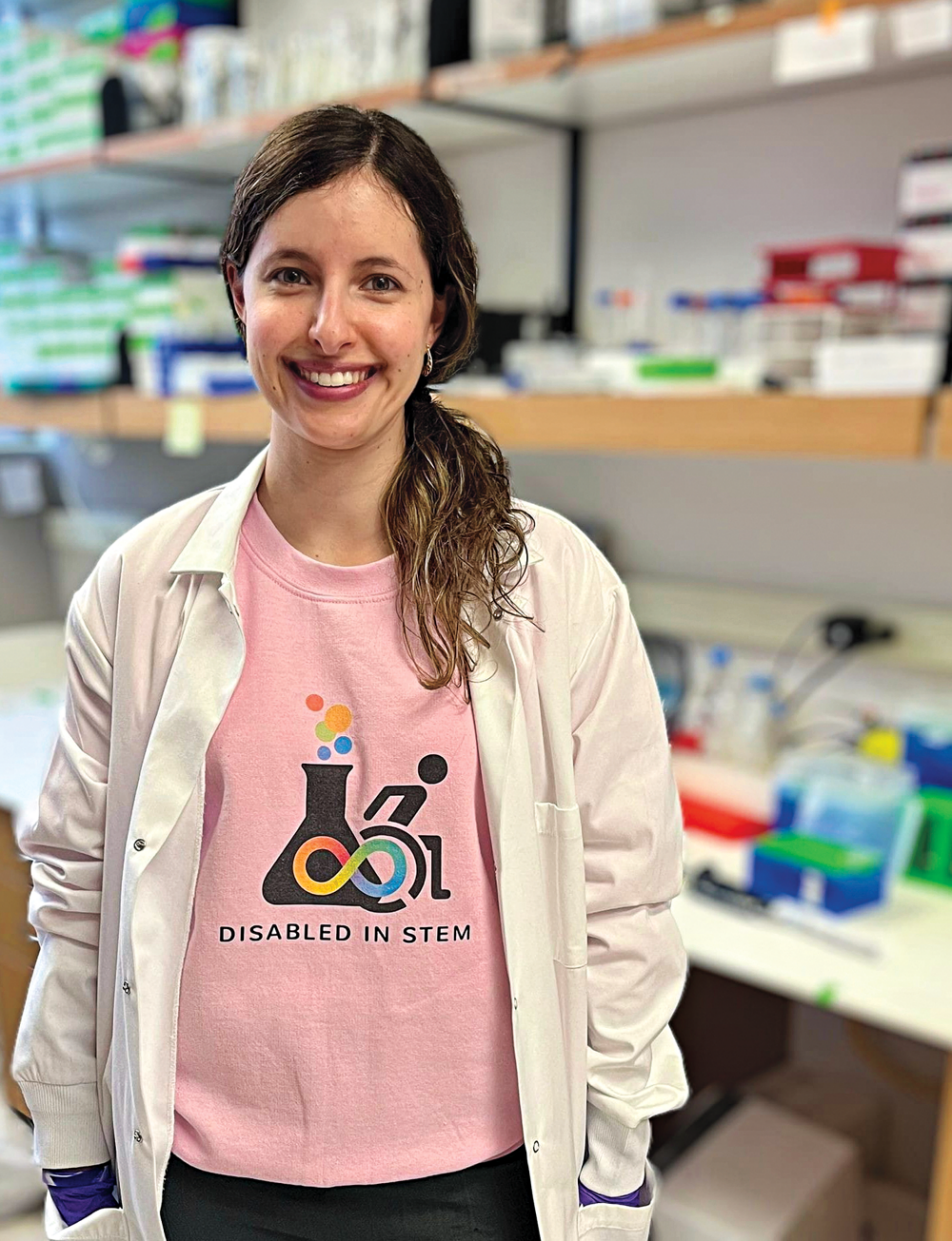
Alyssa Paparella, a cancer and cell biology Ph.D. student at Baylor College of Medicine, founded DisabledInSTEM on X, formerly Twitter, to create a community for disabled scientists. Credit: Courtesy of Alyssa Paparella.

While the Americans with Disabilities Act (ADA) in the U.S. and similar civil rights legislation around the world require newly constructed facilities to be physically accessible, laboratories that predate these laws are not legally required to be retrofitted. Even for newer laboratories, ADA standards apply only to the elements of a building that are fixed or built in, like ramps, bathrooms, and elevators. They don’t consider the accessibility of other things a researcher might use, such as chairs or some scientific equipment. As a result, scientists with disabilities “have to create all this stink just to be able to function in a lab,” Bracher says.

Going through a university’s official accommodation process can be “a massive mental burden,” Travis says. To make matters worse, many disability services offices are geared toward providing office or classroom accommodations and have limited experience helping disabled people in research laboratories.

Furthermore, it can be nerve racking to be the one that has to ask for changes—and the money required to pay for them, says Uma Chatterjee, a neuroscience Ph.D. student at the University of Wisconsin–Madison. Her Ph.D. adviser is supportive when it comes to accommodating her multiple disabilities, but she suspects that not everyone she considered working with would be.

**Figure:**
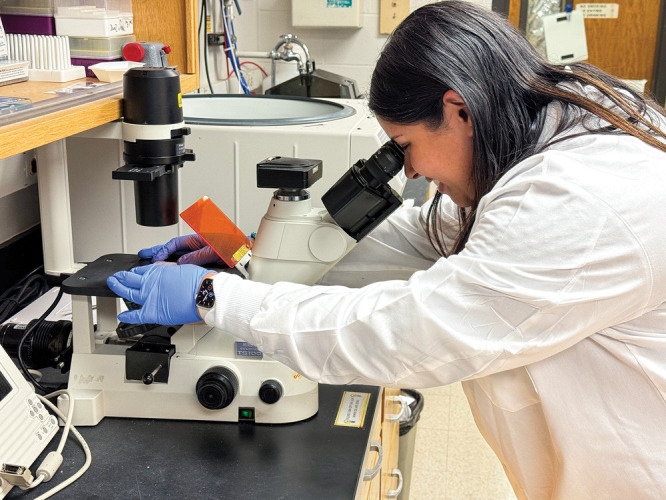
Uma Chatterjee, a neuroscience Ph.D. student at the University of Wisconsin–Madison, says it can be nerve racking to ask for accommodations. Credit: Courtesy of Uma Chatterjee.

It’s perhaps no surprise, then, that many scientists with disabilities say inaccessible laboratories deter disabled individuals from participating in science, technology, engineering, and mathematics (STEM). According to a National Science Foundation report, people with disabilities account for only 3% of the STEM workforce despite making up around 27% of the U.S. adult population. One solution is to minimize barriers so that disabled researchers are “enabled to do their best work in the most comfortable and nonharmful environment,” Travis says.

That involves making lab environments more welcoming to scientists with disabilities, which in the end would be better for everyone, regardless of disability status. The unfortunate reality is that universities and nondisabled scientists don’t consider accessibility in the lab until a disabled individual speaks up, says Alyssa Paparella, a disabled cancer and cell biology Ph.D. candidate at Baylor College of Medicine. “And what does that say about how they are valued?”

## Picturing an accessible lab

What does an accessible lab even look like? Ask five disabled researchers that question, and they’ll likely give five different answers. “It’s hard to think of just this one design that would fit everybody because disability is so varied and diverse,” Paparella says.

That doesn’t mean researchers haven’t tried. In 2010, Brad Duerstock, a professor of engineering practice at Purdue University, began renovating a biology lab on campus with disabled people in mind. “We were able to modify certain corners of it to be more accessible,” he says.

At first, Duerstock and his team focused on making the lab more accommodating to people who use wheelchairs, and he drew from his personal experience. “But then we started thinking about inclusivity—being an inclusively designed lab that would adjust to different people’s needs,” he says.

The result was the Accessible Biomedical Immersion Laboratory, or ABIL. Its main features are a height-adjustable workbench and a wheelchair-accessible sink and fume hood, all located close to one another. Duerstock refers to this setup as an accessible “work triangle.”

Duerstock and his team also adjusted the lab’s eyewash and emergency shower station. “For the emergency shower, the pull to release the water was very high,” Duerstock recalls. So the researchers added a second, lower shower pull that is easier to grab for someone in a wheelchair or with a disability that makes the original pull hard to find or reach.

Of course, making a lab more accessible isn’t cheap, especially when an existing space needs to be renovated. The ABIL project came to fruition only after Duerstock won a $2 million grant from the U.S. National Institutes of Health.

Depending on what needs to be changed, price tags can range from the tens of thousands all the way to the hundreds of thousands of dollars for major modifications, says Ellen Gordon, a laboratory ventilation specialist in Cornell University’s Department of Environment, Health, and Safety.

Although universities occasionally have funding set aside to improve their facilities, the lab’s principal investigator (PI) is more often responsible for much of the cost. In most cases, “you don’t get funding to make your lab more accessible or more efficient,” Duerstock says.

Other disabled scientists have noticed this money gap too. “When we look at the landscape of funding, there’s not really a focus on funding for accessible equipment,” says Blaine Fiss, a postdoctoral researcher in chemistry at Western University who has a disability.

**Figure:**
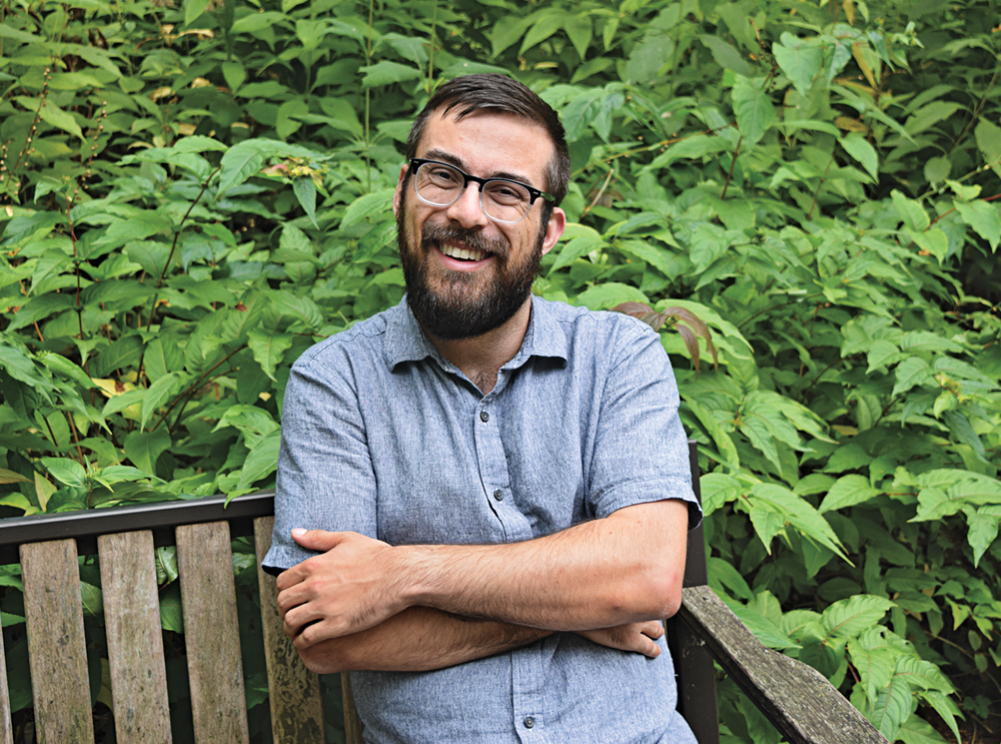
Blaine Fiss, a postdoctoral researcher at Western University, says funding is needed before labs can be made more accessible. Credit: Courtesy of Blaine Fiss.

Smaller pots of money do exist. Paparella, for example, received $1,000 from the International Alliance for Ability in Science, which she put toward buying an electronic pipet that would place less demand on her joints. But that’s not nearly enough to pay for any structural modifications or larger changes that a lab might need.

“Labs are going to remain inaccessible or mostly inaccessible until we get funding to make those physical changes,” Fiss says.

## Better for everyone

Funding issues aside, Duerstock’s ABIL setup demonstrates that making laboratories more accessible for researchers with disabilities is possible. Not only that, but PIs can do a number of things to make laboratories more welcoming, even if they don’t have a specific person with a disability in mind.

Other researchers have been trying to get the message across that making laboratories more accessible is possible. “That’s what I’ve spent my life doing,” says Janet Baum, a program codirector and instructor at the Harvard T. H. Chan School of Public Health. She has coauthored and contributed to numerous books and taught courses on how to design laboratories that are safe and accessible from the get-go for as many people as possible.

Baum says her ultimate goal is “to try and bring up the standard level for all laboratories.” This goal is important not only for people who currently have disabilities but for everyone, as “you never know when someone on staff is going to become disabled,” Baum says. According to the World Health Organization, almost everyone will experience disability at some point in their life.

Accommodating a researcher with a disability doesn’t always have to involve full lab renovations. In addition to making structural changes in the lab, Duerstock equipped ABIL with a talking lab scale, paddle-shaped knobs, and clear signage, all of which can help people with a range of disabilities.

Having accessible equipment or features available for someone who might need them “is going to make them feel welcome to join the space,” Paparella says. They’ll feel less like a burden, she adds, because they won’t have to say, “You need to spend all this money in order to have me.”

And making a lab more accessible doesn’t help just disabled researchers. Paparella bought the electronic pipet to help get through her experiments with less joint pain, but when others in the lab borrow it, they note how their wrist and thumb feel much better at the end of the day. “People didn’t realize that having these small adaptations would make such a difference in their life,” she says.

Similarly, Fiss has cerebral palsy, which affects his balance and coordination. As a grad student, he struggled to load samples in the nuclear magnetic resonance spectrometer since doing so required stepping on and off a flimsy step stool. His department replaced the step stool with a sturdier set of wooden stairs so he could more safely load his samples.

None of his nondisabled colleagues ever complained about the stairs being there. “These changes, whether they are for visible disabilities or invisible disabilities, are ultimately going to benefit everyone,” he says.

## Making it happen

Every lab and every person is different, but whatever the accommodation request, resources exist for fulfilling it.

The Royal Society of Chemistry recently released a report highlighting some common barriers that chemists with disabilities face and what the scientific community can do about them. According to Travis, the disability and accessibility specialist, the society plans to release more resources pertaining to lab accessibility over the next year.

Both disabled and nondisabled scientists can also contact the American Chemical Society Committee on Chemists with Disabilities (CWD) if they’re unsure what types of lab accommodations are possible. ACS publishes C&EN but is not involved in editorial decisions. “You can email and then you could get a perspective from a chemist that has been through” the experience of receiving lab accommodations, says Amie Norton, a postdoctoral researcher in entomology at Kansas State University and member of CWD. If that group doesn’t have an answer, it can find someone who does.

Social media is another resource. On platforms like X, formerly Twitter, many scientists with disabilities talk openly about how they navigate accessibility issues in STEM. For example, Paparella runs the account @DisabledSTEM, which has enabled disabled scientists to meet one another, commiserate, and share the accommodations they are seeking. “It’s nice to have that community, even if it’s virtual, to know you’re not alone,” she says.

**Figure:**
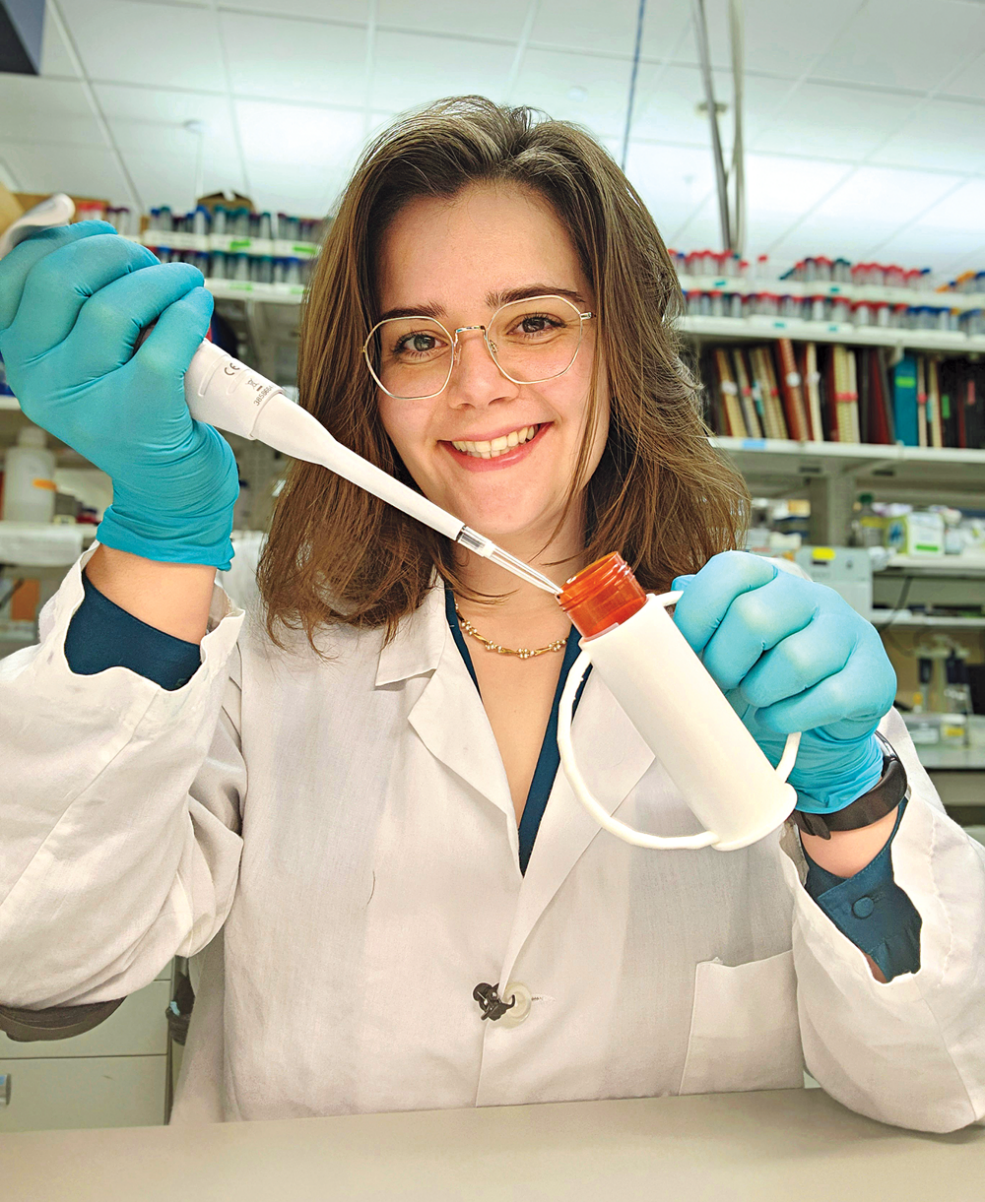
Katharine Hubert, a genetics Ph.D. candidate at the University of Wisconsin–Madison, recently launched #LabdaptationsIn3D, a project in which she shares 3D templates of accessible lab tools. Her first design is a 50 mL conical tube holder. Credit: Courtesy of Katharine Hubert.

Meanwhile, Katharine Hubert, a genetics Ph.D. candidate at the University of Wisconsin–Madison, shares how she accommodates her own disability on her account @cripple_vs_STEM and through the hashtag #Labdaptations. Hubert recently launched #LabdaptationsIn3D, a project in which she shares 3D templates of accessible lab tools she has designed so that anyone with a 3D printer can make the tools for themselves. Her first design is a 50 mL conical tube holder.

For bigger changes, there are experts, like Baum and Gordon, who have experience with ensuring laboratories are both accessible and safe. But to truly make science accessible, scientists need to consider more than just altering physical spaces and modifying lab equipment. “It’s not just about accommodating this group. It’s about making sure they have a place,” Norton says.

“People who are not disabled tend to see disability in such a negative connotation,” Paparella says. Their ableism, whether implicit or explicit, “really affects how they perceive me and what my opportunities are.”

Bracher remembers people asking him why he didn’t just choose to do computational chemistry; they couldn’t fathom how he could succeed in the lab. “A terrible way to manage someone disabled is to steer them into something they don’t want to do. But it happens all the time,” he says.

That’s not only bad for individuals with disabilities; it’s bad for STEM. “People with disabilities have a unique perspective,” Norton says. “It’s important to incorporate that group.”

## A note about language

- People with disabilities are incredibly varied, and the choice of language to describe them is also varied.
- Two common choices are people-first (e.g., “person with a disability”) and identity-first (e.g., “disabled person”) language.
- Some people prefer people-first language because it emphasizes that their condition does not define them. Identity-first proponents, meanwhile, say that their disability is part of their identity and that people-first language can imply that disability is something to be ashamed of.
- In this story, when we describe someone we interviewed, we defer to the language they asked us to use. For more general usage, we alternate between people-first and identity-first language.

**Sources:**U.S. Centers for Disease Control and Prevention, ACS Inclusivity Style Guide.

*Krystal Vasquez is an associate editor at*Chemical & Engineering News*, the independent news outlet of the American Chemical Society. A version of this story appeared inC&EN.*

